# The pattern of dyslipidemia among Somali type 2 diabetic patients: a cross-sectional study

**DOI:** 10.1186/s40001-022-00882-x

**Published:** 2022-11-20

**Authors:** Gökhan Alıcı, Ömer Genç

**Affiliations:** 1 Department of Cardiology, Somalia Mogadishu Türkiye Recep Tayyip Erdogan Training and Research Hospital, Mogadishu, Somalia; 2 Department of Cardiology, İstanbul Başakşehir Çam and Sakura City Hospital, İstanbul, Turkey

**Keywords:** Diabetes mellitus, Dyslipidemia, Somalia, LDL-C, Atherogenic dyslipidemia

## Abstract

**Background:**

Diabetes mellitus (DM) is a major public health concern. This study aims to determine frequency, pattern, and potential determinants of dyslipidemia among adults with type 2 DM (T2DM) at Somalia’s only diabetes outpatient clinic.

**Methods:**

Five hundred twenty-nine consecutive patients with T2DM who applied to our outpatient clinic between January 2020 and June 2020 were included in this cross-sectional hospital-based study. Demographic characteristics of participants, including lipid panel, were extracted from the registry system. Correlation analysis was performed between lipid profile and related parameters. Multivariate binary logistic regression models were used to identify independent determinants of dyslipidemia for further analysis.

**Results:**

The overall population’s mean age was 51.9 ± 12.2 years, with 177 (33.5%) males. Total and atherogenic dyslipidemias were found in 92.8% and 24.8%, respectively. The most common isolated pattern of dyslipidemia was high non-high-density lipoprotein cholesterol (non-HDL-C) (82.8%), followed by high low-density lipoprotein cholesterol (LDL-C) (72.6%), high total cholesterol (TC) (54.3%), and low HDL-C (48.3%). Females were found to have a higher prevalence of high TC (63.4% vs. 54.2%, *p* = 0.043) and lower HDL-C (57.4% vs. 46.3%, *p* = 0.016). High LDL-C with low HDL-C was the most common pattern among combined type dyslipidemias (18.1%), followed by high LDL-C with high triglyceride (TG) (17.8%), as well as low TG with low HDL-C (3.6%). Females had a higher proportion of high LDL-C with low HDL-C than males (20.3% vs. 13.6%, *p* = 0.036). Age, gender, body mass index, central obesity, spot urinary proteinuria, fasting blood glucose, poor glycemic control, creatinine, and Hs-CRP were all associated with different dyslipidemia patterns in multivariate logistic regression analyses.

**Conclusions:**

We found that the prevalence of dyslipidemia, especially atherogenic patterns, was extremely high among Somali T2DM patients. An enhanced health policy should, therefore, be established to detect, treat and prevent dyslipidemia.

**Supplementary Information:**

The online version contains supplementary material available at 10.1186/s40001-022-00882-x.

## Background

Diabetes mellitus (DM) is a common endocrine-metabolic disorder that is the leading cause of morbidity and mortality across the globe [1]. Chronic hyperglycemia among patients with DM contributes to the development of cardiovascular disease by hastening the atherosclerotic process [2]. Furthermore, because of the crucial regulatory effects of insulin on lipid metabolism, dyslipidemia is a well-known manifestation of uncontrolled DM [3]. Diabetic dyslipidemia is mainly characterized by higher plasma triglyceride (TG), lower high-density lipoprotein cholesterol (HDL-C), and higher low-density lipoprotein cholesterol (LDL-C) concentrations [4], posing a substantial risk in the pathogenesis of early atherosclerosis independent of other risk factors [5]. In addition, more than half of patients with type 2 diabetes (T2DM) die from coronary artery disease, which is caused primarily by atherogenic dyslipidemia [6]. Moreover, effective therapy of dyslipidemia may dramatically decrease the development of atherosclerosis [7]. Controlling diabetic dyslipidemia, a reversible risk factor for atherosclerosis, is, therefore, a critical step.

Due to the conditions of the civil war, Somalia, one of the least developed countries in the region, has no social security system or state policy in the field of health [8]. The health system is mostly run by private health institutions and is not subject to state oversight. Besides, access to medications and diagnostic modalities required for the follow-up and treatment of chronic diseases in the country is extremely difficult and unequal [9]. Except for Somali refugees in emigrating countries, there is little information on DM and dyslipidemia in Somalia, and no studies regarding the prevalence and pattern of diabetic dyslipidemia exist [10–13]. Our study may be a significant step toward filling a gap in the existing literature. Based on this information, the study’s objectives were twofold: (1) to investigate the prevalence and pattern of dyslipidemia among T2DM patients in Somalia, and (2) to identify determinants that may be associated with various dyslipidemia subgroups.

## Methods

### Study design and setting

This retrospective, cross-sectional study was carried out at the Somali–Turkey Training and Research Hospital in Mogadishu, the country’s largest tertiary care center. The institution is the only location where advanced investigations and treatments, including the only diabetes outpatient clinic in the country, are accessible. The study consecutively included 529 individuals aged 18 years or older who were followed up on or treated for DM between January 1 and June 1, 2020. Those were excluded from the analysis if they were under 18 (*n* = 13) or had gestational DM (*n* = 3), steroid-induced DM (*n* = 3), thyroid disease (*n* = 8), acute/chronic liver failure (*n* = 3), systemic/local infection (*n* = 4), congestive heart failure (*n* = 8), pregnancy (*n* = 4), or had taken a lipid-lowering agent in the previous month (*n* = 21).

### Demographic, clinical, and laboratory parameters

The hospital database was reviewed to identify those who met the eligibility as well as to collect information from patients included in the present study for further analysis. As part of the routine examination, laboratory parameters were acquired on an empty stomach. All biochemical measurements were performed using an automated chemistry analyzer and fully prepared reagent kits (Mindray BS 2000 M, CHINA) in accordance with the manufacturer’s standardized protocol. Total cholesterol (TC) and TG levels were determined using the cholesterol oxidase/peroxidase and glycerol phosphate kinase methods, respectively. The phosphotungstate precipitation method was used to determine HDL-C, and the Friedewald formula was utilized to calculate LDL-C [14]. The ion exchange resin method was employed to determine glycosylated hemoglobin (HbA1c) in EDTA blood. Body mass index (BMI), calculated as weight (kg) divided by height (m) squared, was classified as underweight (< 18.5 kg/m^2^), normal (18.5–24.9 kg/m^2^), overweight (25.0–29.9 kg/m^2^), and obese (≥ 30.0 kg/m^2^) by the World Health Organization [15]. The waist circumference (WC) was measured twice at the midpoint between the lower edge of the costal arch and the upper edge of the iliac crest, immediately after standing up straight and exhaling, and the average was recorded. A diabetes education nurse took the measurements after at least 8 h of fasting. Central obesity was defined as a WC of 94 cm in men and 80 cm in women. UriSed^®^ 3 PRO (77 Elektronika, Hungary), a fully automated urine sediment analyzer connected to the LABUMAT 2 urine strip reader, was utilized to measure qualitative protein analysis in urine.

### Diabetes mellitus and dyslipidemia

According to current World Health Organization criteria [16], DM was diagnosed in those who fulfilled at least one of the following criteria; fasting blood glucose (FBG) values of ≥ 126 mg/dL, 2-h post-load plasma glucose ≥ 200 mg/dl, HbA1c ≥ 6.5% or a random blood glucose ≥ 200 mg/dL in the presence of signs and symptoms. Poor glycemic control was defined as HbA1c > 7%. After at least 8 h of fasting, dyslipidemia was defined as the presence of at least one of the following criteria, taking into account the criteria of the third National Cholesterol Education Program Adult Treatment Panel (NCEP ATP III) report [6]; TC ≥ 200 mg/dL, TG ≥ 150 mg/dL, LDL-C ≥ 100 mg/dL, Non-HDL-C ≥ 130 mg/dL, HDL-C limit < 40 mg/dL for men and < 50 mg/dL for women. Isolated dyslipidemia is defined as meeting only one of the above criteria, whereas mixed or combined dyslipidemia is defined as the presence of any two of the dyslipidemias. LDL-C ≥ 100 mg/dL and TG ≥ 150 mg/dL were considered atherogenic, along with HDL-C < 40 mg/dL for men and < 50 mg/dL for women. Based on registry data, hypertension was defined as being on anti-hypertensive therapy or having a systolic blood pressure of ≥ 140 mmHg and/or a diastolic blood pressure of ≥ 90 mmHg.

### Sample size

During the planning phase of the study, a power analysis was performed to ensure a sufficient number of participants. The following cross-sectional study formula was used to calculate sample size for prevalence: *n* = [*Z*^2^ *P* (1 − *P*)]/*d*^2^] [17].

The estimated prevalence is denoted by *P*, the statistical level of confidence by *Z*, and the precision by *d*. The confidence level was assumed to be 95%, with a precision of 5%. (Corresponding to *Z* value of 1.96). The only study on the frequency of dyslipidemia in Somalia was conducted on Somali refugees and the prevalence was found to be 18.1% [13]. Therefore, the number of patients should have been at least 228. However, since our study was conducted on T2DM with more frequent dyslipidemia, we arbitrarily planned to recruit roughly twice as many participants as the number calculated.

### Statistical analysis

The analysis was conducted using Statistical Package for Social Sciences (SPSS), version 20 (SPSS Inc., Chicago, IL, USA). Continuous variables were tested for normality distribution using an analytical method (Kolmogorov–Smirnov test) and visual methods (histograms and probability plots). Continuous variables were expressed as mean ± standard deviation or median (interquartile range; IQR_25–75_), whereas categorical variables were expressed as numbers (*n*) and percent (%), as appropriate. The independent sample *t* test or the Mann–Whitney *U* test was used to analyze continuous variables. Categorical variables were compared using the χ^2^-test or Fisher’s exact test. The One-way ANOVA was used for comparing serum levels of a single lipid parameter across six different age groups. Spearman correlation or Pearson correlation coefficient analysis was utilized to investigate the relationship between age, BMI, HbA1c, FBG, WC, and lipid parameters and ratios. Multivariate binary logistic regression models were used to identify independent determinants of dyslipidemia for the whole study population and genders, separately. The Hosmer–Lemeshow test was performed to evaluate the goodness-of-fit in the regression analysis. Internal correlation analysis (multicollinearity) was performed by testing whether variance inflation factor < 3, condition index < 15, and variance proportions < 0.6 were achieved using numerical expressions of individual lipid parameters. Odds ratio (OR) and 95% confidence interval (CI) were quantified for each independent variable except smoking, which was not calculated in the regression analysis for men and women due to the insufficient number of events. All analyses used two-sided tests with an overall significance level (alpha) of 0.05.

## Results

### Baseline characteristics

The study included 529 consecutive patients with T2DM [177 (33.5%) men, 51.9 ± 12.2 mean age]. The mean HbA1c level was 9.7 ± 2.2. Three hundred forty (64.2%) of the study population received only oral anti-diabetic (OAD) treatment, 35 (6.6%) received only insulin, and 153 (28.9%) received both OAD and insulin. Males were more likely to have a higher BMI than females (33.1 ± 2.8 vs. 31.4 ± 2.3, *p* < 0.001). Only 14 (2.6%) of patients were smokers, with males smoking at a higher rate than females [13 (7.3%) vs. 1 (0.3%), *p* < 0.001]. Females had higher mean HDL-C cholesterol levels than males (46.9 ± 11.4 vs. 42.3 ± 11.3, *p* < 0.001). The mean values of TC, LDL-C, Non-HDL-C, and TG levels did not differ between genders. The vast majority of patients (*n* = 468, 88.5%) had inadequate glycemic control. Table 1 summarizes the overall study population’s demographics, clinical and laboratory results, medications, and chronic diseases.

**Table 1:** Baseline characteristics of the overall population

Variable	All (*N* = 529)	Male (*n* = 177)	Female (*n* = 352)	*p* value*
Age (year), mean ± SD	51.9 ± 12.2	52 ± 12.6	51 ± 12.1	0.685
Age category, *n* (%)				
24–34	40 (7.6)	14 (7.9)	26 (7.4)	0.442
35–44	96 (18.1)	33 (18.6)	63 (17.9)	
45–54	176 (33.3)	49 (27.7)	127 (36.1)	
55–64	130 (24.6)	51 (28.8)	79 (22.4)	
65–74	61 (11.5)	22 (12.4)	39 (11.1)	
≥ 75	26 (4.9)	8 (4.5)	18 (5.1)	
BMI (kg/m^2^), mean ± SD	31.9 ± 2.8	33.1 ± 3.2	31.4 ± 2.3	**< 0.001**
Categorical BMI, *n* (%)				
Normal	1 (0.2)	0 (0)	1 (0.3)	0.576
Overweight	55 (10.4)	21 (11.9)	34 (9.7)	
Obese	473 (89.4)	156 (88.1)	317 (90.1)	
Waist circumference (cm), mean ± SD	101.8 ± 7.3	102.6 ± 8.2	101.4 ± 6.9	0.111
Central obesity, *n* (%)	492 (93.0)	140 (79.1)	352 (100.0)	**< 0.001**
Smoking, *n* (%)	14 (2.6)	13 (7.3)	1 (0.3)	**< 0.001**
Hypertension, *n* (%)	258 (86.4)	149 (84.2)	309 (87.8)	0.251
CKD *n* (%)	60 (11.3)	24 (13.6)	36 (10.2)	0.254
Hemoglobin (g/dL), mean ± SD	13.78 ± 1.85	13.76 ± 1.81	13.79 ± 1.87	0.877
Ure (mg/dL), median (IQR)	30 (19)	30 (17)	30 (15)	**0.029**
Creatinine (mg/dL), median (IQR)	0.9 (0.38)	0.91 (0.30)	0.90 (0.35)	**0.013**
Na (meq/L), mean ± SD	137.6 ± 4.2	137.7 ± 4.4	137.6 ± 4.0	0.779
K (meq/L), median (IQR)	4 (0.7)	3.9 (0.8)	4 (0.7)	0.319
HDL-C (mg/dL), mean ± SD	45.3 ± 11.6	42.3 ± 11.3	46.9 ± 11.4	**< 0.001**
LDL-C (mg/dL), mean ± SD	129.2 ± 36.9	128.9 ± 38.6	129.3 ± 36.1	0.897
TC (mg/dL), mean ± SD	216.3 ± 49.5	213.2 ± 52.3	217.9 ± 48.0	0.311
TG (mg/dL), median (IQR)	170.6 ± 96.4	169.7 ± 96.9	170.9 ± 96.3	0.882
Non-HDL-C (mg/dL), mean ± SD	170.9 ± 48.1	170.9 ± 49.9	171.0 ± 47.3	0.995
Fasting glucose (mg/dL), median (IQR)	268 (168)	271 (172)	265 (164)	0.951
hs-CRP (mg/L), median (IQR)	14 (15)	12 (15)	15 (16)	0.106
HbA1c (%), mean ± SD	9.7 ± 2.2	9.8 ± 2.4	9.6 ± 2.1	0.277
Poor glycemic control, *n* (%)	468 (88.5)	158 (89.3)	310 (88.1)	0.684
Medical, *n* (%)				
Insulin	35 (6.6)	16 (9)	19 (5.4)	
OAD	340 (64.2)	109 (61.6)	231 (65.8)	0.262
Both	153 (28.9)	52 (29.4)	101 (28.8)	

Data are presented as numbers and percentages (%), mean (standard deviation), or median (interquartile range). *p* value was calculated using the Independent Samples *t* test or the Mann–Whitney *U* test for continuous variables and the Chi-Square test or the Fisher’s exact test for categorical variables as appropriate. *HDL-C* high-density lipoprotein cholesterol, *LDL-C* low-density lipoprotein cholesterol, *TC* total cholesterol, *TG* triglycerides, *hs-CRP* high-sensitivity C-reactive protein, *OAD* oral anti-diabetes drug, *BMI* body mass index, *HbA1c* hemoglobin A1c, *CKD* chronic kidney disease

*p value < 0.05 was considered significant

### Dyslipidemia patterns

**Fig. 1:**
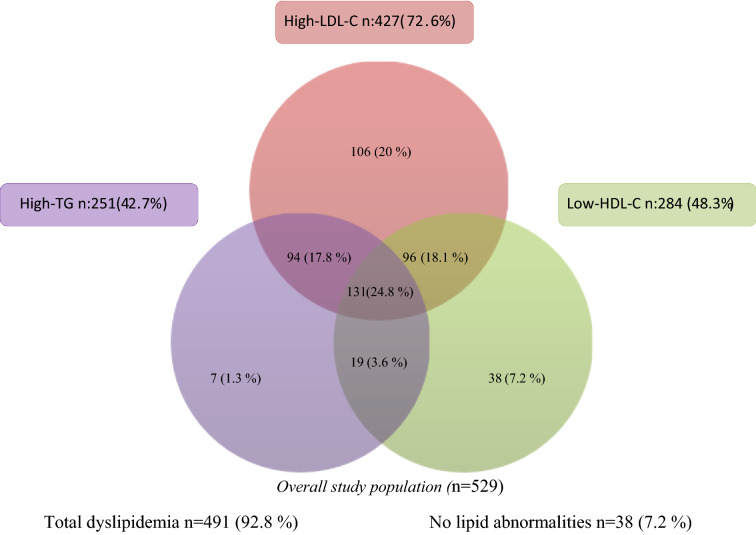
Venn diagram showing the overlapping of the individual components of atherogenic dyslipidemia criteria. *HDL-C* high-density lipoprotein, *LDL-C* low-density lipoprotein, *TG* triglycerides

The prevalence of total dyslipidemia was as 92.8%, with no significant difference between the genders (94.4% vs. 92.0%, *p* = 0.333). One-hundred thirty-one (24.8%) participants met the criteria for atherogenic dyslipidemia, which was similar between men and women (*p* = 0.302). Figure 1 depicts a Venn diagram detailing the components of atherogenic dyslipidemia criteria.

**Fig. 2:**
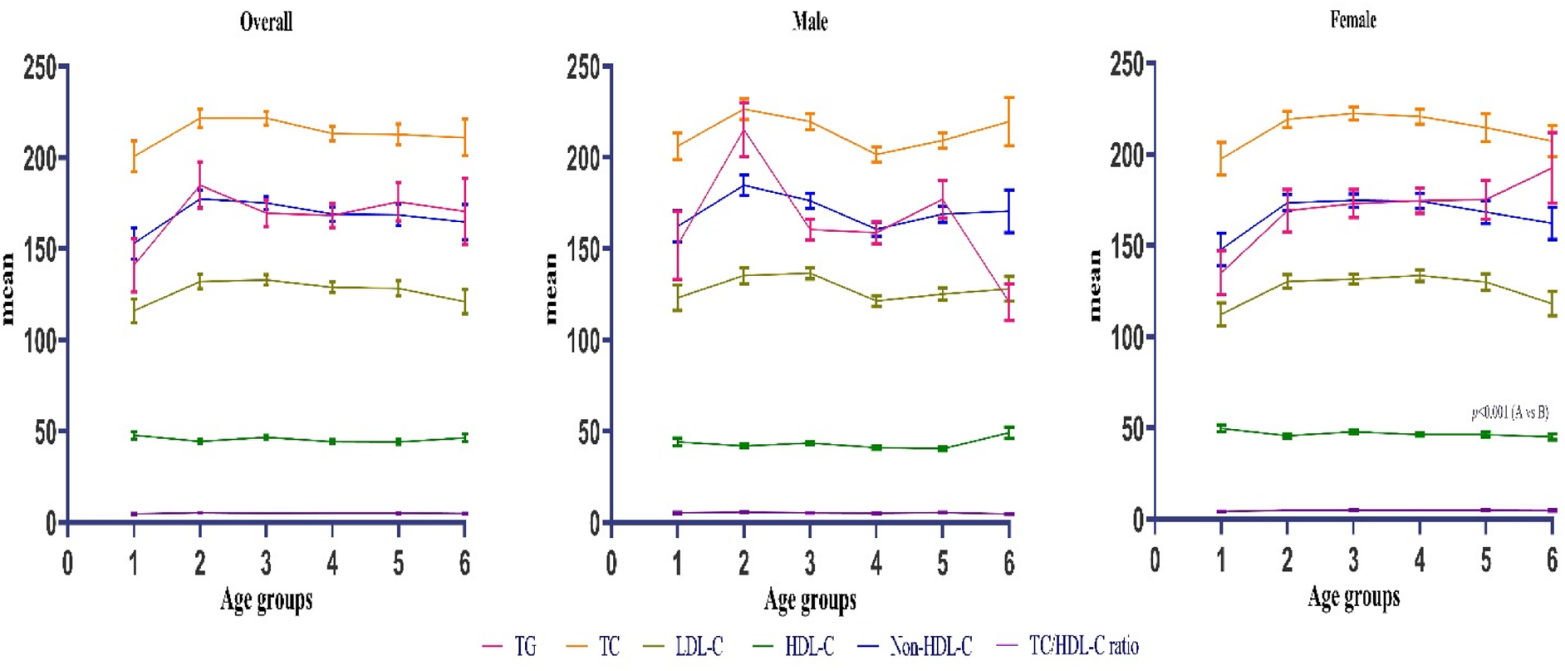
Age- and sex-specific mean values of serum lipid parameters and TC/HDL-C ratio in the study population. One-way ANOVA was used to compare serum levels of a single lipid parameter among six different age groups. In age groups, 1 indicates those between the ages of 24 and 34, 2 35–44, 3 45–54, 4 55–64, 5 65–74, and 6 75 and over. All single lipid parameters have a statistically insignificant distribution among age groups (*p* > 0.05, for all). *The mean* reflects serum lipid parameter concentrations (mg/dL) and TC to HDL-C ratio values. *TG* triglycerides, *TC* total cholesterol, *HDL-C* high-density lipoprotein cholesterol, *LDL-C* low-density lipoprotein cholesterol

The most prevalent dyslipidemia was high non-HDL-C (*n* = 438, 82.8%), followed by high LDL-C (*n* = 427, 72.6%), high TC (*n* = 319, 54.3%), low HDL-C (*n* = 284, 48.3%), and high TG (*n* = 251, 42.7%). Women were found to have higher rates of high TC and lower levels of HDL-C than men (*p* = 0.043 and *p* = 0.016, respectively). The prevalences of isolated dyslipidemias were as follows: high LDL-C (*n* = 106, 20.0%), low HDL-C (*n* = 38, 7.2%), and high TG (*n* = 7, 1.3%), respectively. Of the mixed-type dyslipidemias, the coexistence of high LDL-C and low HDL-C was found to be the most common (*n* = 96, 18.1%), which was at a higher rate in females than in males [71 (20.3%) vs. 24 (13.6%), *p* = 0.036]. Other combined dyslipidemias were high LDL-C and TG (*n* = 94, 17.8%) and high TG and low HDL-C (*n* = 19, 3.6%), respectively, with no statistically significant gender difference. Table 2 shows the distribution of dyslipidemia patterns in patients by gender. No difference was observed among age groups in terms of single lipid parameters and TC/HDL ratio. Except for HDL-C, which was higher in females than in males (*p* < 0.001), lipid parameters were comparable. Age- and sex-stratified serum lipid parameters and TC/HDL-C ratio are shown in Fig. 2.

**Table 2:** Pattern of dyslipidemia

Lipid abnormality	All *n* (%)	Male *n* (%)	Female *n* (%)	*p* value*
High TC (TC ≥ 200 mg/dl), n (%)	319 (54.3)	96 (54.2)	223 (63.4)	**0.043**
High TG (TG ≥ 150 mg/dl), n (%)	251 (42.7)	82 (46.3)	169 (48.0)	0.714
High LDL-C (LDL-C ≥ 100 mg/dl), n (%)	427 (72.6)	144 (81.4)	283 (80.4)	0.792
High Non-HDL-C (Non-HDL-C ≥ 130 mg/dl), n (%)	438 (82.8)	151 (85.3)	287 (81.5)	0.277
Low HDL-C (HDL-C < 40/50 mg/dl), n (%)	284 (48.3)	82 (46.3)	202 (57.4)	**0.016**
TC/HDL-C ratio	5.01 ± 1.50	5.29 ± 1.57	4.87 ± 1.45	**0.003**
Isolated single parameter dyslipidemia, *n* (%)				
LDL-C ≥ 100 mg/dl and TG < 150 mg/dl and HDL-C > 40/50 mg/dl	106 (20.0)	47 (26.6)	59 (16.8)	**0.008**
LDL-C < 100 mg/dl and TG ≥ 150 mg/dl and HDL-C > 40/50 mg/dl	7 (1.3)	4 (2.3)	3 (0.9)	0.181
LDL-C < 100 mg/dl and TG < 150 mg/dl and HDL-C < 40/50 mg/dl	38 (7.2)	14 (7.9)	24 (6.8)	0.646
Mixed Two Parameter Dyslipidemias, n (%)				
LDL-C ≥ 100 mg/dl and TG ≥ 150 mg/dl and HDL-C ≥ 40/50 mg/dl	94 (17.8)	33 (18.6)	60 (17.1)	0.371
LDL-C < 100 mg/dl and TG ≥ 150 mg/dl and HDL-C < 40/50 mg/dl	19 (3.6)	5 (2.8)	14 (4.0)	0.344
LDL-C ≥ 100 mg/dl and TG < 150 mg/dl and HDL-C < 40/50 mg/dl	96 (18.1)	24 (13.6)	71 (20.3)	**0.036**
Total Dyslipidemia, *n* (%)				
LDL-C ≥ 100 mg/dl and/or TG ≥ 150 mg/dl and/or HDL-C < 40/50 mg/dl Atherogenic Dyslipidemia, n (%)	491 (92.8)	167 (94.4)	324 (92.0)	0.333
LDL-C ≥ 100 mg/dl and TG ≥ 150 mg/dL and HDL-C < 40/50 mg/dl	131 (24.8)	39 (22)	92 (26.1)	0.302

Data are presented as numbers and percentages (%) or mean (standard deviation). *p* value was calculated using the Independent Samples *t* test for continuous variables and the Chi-Square test or the Fisher’s exact test for categorical variables as appropriate. *HDL-C* high-density lipoprotein cholesterol, *LDL-C* low-density lipoprotein cholesterol, *TC* total cholesterol, *TG* triglycerides

*p value < 0.05 was considered significant

### Correlates and determinants of dyslipidemia

Age was weakly correlated positively with BMI (*r* = 0.096). There was a positive correlation between WC and BMI (*r* = 0.683), as well as between HbA1c and FBG (*r* = 0.486). Furthermore, there was a positive correlation between FBG and TG (*r* = 0.132), LDL-C (*r* = 0.145), non-HDL-C (*r* = 0.164), TC (*r* = 0.163), and TC/HDL-C ratio (*r* = 0.098), but no significant correlation was found between HbA1c and lipid parameters. Table 3 shows the bivariate correlation analysis of lipids and some other parameters.

**Table 3:** Correlation among age, BMI, HgA1c, FBG, WC and lipid parameters and ratio

Variables	1	2	3	4	5	6	7	8	9	10	11
1.Age	–	0.096*	− 0.031	− 0.018	− 0.031	0.033	0.000	− 0.027	−0.001	− 0.008	0.000
2.BMI		–	− 0.010	− 0.048	0.683***	0.062	0.065	− 0.028	0.069	0.061	0.085
3.HgA1c			–	0.486***	− 0.014	− 0.031	0.022	0.081	0.039	0.057	− 0.016
4.FBG				–	− 0.059	0.132**	0.145**	0.047	0.164**	0.163**	0.098*
5.WC					–	0.018	0.080	0.032	0.081	0.086	0.044
6.TG						–	0.304***	− 0.273***	0.413***	0.338***	0.509***
7.LDL-C							–	0.065	0.896***	0.887***	0.582***
8.HDL-C								–	− 0.004	0.231***	− 0.634***
9.Non-HDL-C									–	0.972***	0.715***
10.TC										–	0.547***
11.TC/HDL-C											–

*HDL-C* high-density lipoprotein cholesterol, *LDL-C* low-density lipoprotein cholesterol, *TC* total cholesterol, *TG* triglycerides, *BMI *body mass index, *HbA1c* hemoglobin A1c, *WC* waist circumference, *FBG* fasting blood glucose

*Refers to *p* value of < 0.05

**Refers to *p* value of < 0.01

***Refers to *p* value of < 0.001

Age, gender, high BMI, and central obesity were all independently associated with hypertriglyceridemia. High TC was related to FBG, poor glycemic control, creatinine, and Hs-CRP. Proteinuria by spot urine sampling and gender were independent determinants of low HDL-C. Moreover, FBG and creatinine were linked to high non-HDL-C, whereas Hs-CRP was the only independent predictor of high LDL-C. Table 4 displays multivariate logistic regression analyses to determine the independent determinants of dyslipidemias.

**Table 4:** Multivariable logistic regression analyses of the whole study population for predictors of the different types of dyslipidemia

Variables	High TG OR (95% CI)	*p* value	High TC OR (95% CI)	*p* value	Low HDL-C (OR) (95% CI)	*p* value	High LDL-C	High Non-HDL-C		
							OR (95% CI)	*p* value	OR (95% CI)	*p* value
Age	1.03 (1.01–1.05)	**0.013**	1.00 (0.98–1.02)	0.882	1.01 (0.99–1.03)	0.257	0.99 (0.97–1.02)	0.691	0.99 (0.96–1.02)	0.508
Male gender	0.43 (0.24–0.79)	**0.006**	0.83 (0.46–1.51)	0.546	0.49 (0.27–0.87)	**0.015**	1.41 (0.62–3.21)	0.415	1.30 (0.56–3.00)	0.543
BMI	1.15 (1.04–1.26)	**0.007**	1.08 (0.98–1.19)	0.141	0.96 (0.87–1.05)	0.337	1.07 (0.94–1.21)	0.325	1.09 (0.95–1.25)	0.208
Central obesity	0.25 (0.08–0.79)	**0.019**	1.06 (0.33–3.42)	0.921	0.96 (0.32–2.95)	0.948	1.94 (0.50–7.62)	0.340	0.64 (0.13–3.23)	0.589
Smoking	5.12 (0.93–28.13)	0.060	0.74 (0.16–3.34)	0.690	2.31 (0.49–10.85)	0.289	1.52 (0.16–14.25)	0.713	0.94 (0.10–8.55)	0.955
Proteinuria	1.80 (0.96–3.38)	0.066	1.08 (0.56–2.10)	0.814	0.49 (0.26–0.91)	**0.024**	1.65 (0.70–3.89)	0.258	1.30 (0.54–3.13)	0.551
Fasting plasma glucose	1.00 (1.00–1.00)	0.104	1.00 (1.00–1.01)	**0.016**	1.00 (0.99–1.00)	0.952	1.00 (1.00–1.01)	0.068	1.00 (1.00–1.01)	**0.017**
Poor glycemic control	1.14 (0.51–2.51)	0.752	0.32 (0.13–0.84)	**0.020**	0.89 (0.40–1.97)	0.776	0.60 (0.20–1.77)	0.356	0.59 (0.19–1.79)	0.347
Creatinine	0.88 (0.68–1.14)	0.344	0.45 (0.25–0.82)	**0.009**	1.34 (0.97–1.85)	0.073	0.79 (0.60–1.00)	0.082	0.68 (0.50–0.93)	**0.015**
Hypertension	1.15 (0.51–2.57)	0.737	0.88 (0.40–1.97)	0.760	0.65 (0.30–1.41)	0.276	1.69 (0.66–4.33)	0.279	1.49 (0.55–4.05)	0.439
Hs-CRP	1.00 (0.99–1.02)	0.743	0.98 (0.96–0.99)	**0.015**	1.02 (0.99–1.03)	0.085	0.98 (0.96–0.99)	**0.006**	0.99 (0.97–1.01)	0.370

Values are presented as OR (95% CI). O*R* = Odds ratio, CI = Confidence Interval

Coding of categorical variables-Gender: female = 0, male = 1; Smoking: non-smoke*r* = 0, current smoke*r* = 1; Proteinuria:absent = 0, present = 1;Glycemic control: good (HbA1c% < 7) = 0,poor (HbA1c% > 7) = 1; Hypertension: normotensive = 0, hypertensive = 1; Fasting plasma glucose, Age, BMI, Hs-CRP, Creatinine, waist circumference were continuous variables

When the regression analysis was stratified by gender; In men (Additional file 1: Table S1), there was an independent association only between FBG and high TC. In women (Additional file 1: Table S2), age, BMI, and FBG were all independent predictors of hypertriglyceridemia. Poor glycemic control and creatinine were linked to high TC. Low HDL-C levels were related to central obesity and proteinuria in the urine. Only Hs-CRP was linked to high LDL-C, whereas creatinine and hypertension were independent predictors of high non-HDL-C.

## Discussion

This is the first large epidemiological report in Somalia that focuses on the distribution of diabetic dyslipidemia, as well as the clinical and demographic characteristics of T2DM. Our study's main findings were as follows; (i) diabetes was poorly controlled, and accordingly, rates of diabetic dyslipidemia were extremely high, (ii) atherogenic dyslipidemia, which is important in the pathophysiology of cardiovascular disease, was also prevalent (24.8%), (iii) The dominant component of the dyslipidemia patterns was high LDL-C, which has a proven atherogenic effect, (iv) various dyslipidemia patterns were found to be associated with age, gender, BMI, central obesity, spot urine proteinuria, FBG, poor glycemic control, creatinine, and Hs-CRP.

Some studies conducted in countries, where Somalis live as refugees (Finland and the United States-based) where Somalis live as refugees (Finland and the United States-based), have found higher rates of obesity, sedentary lifestyle, diabetes, hypertension, and hyperlipidemia, all of which are cardiovascular risk factors [10, 11, 13]. For example, in a study of 1007 participants, high prevalence rates of obesity (68%), dyslipidemia (18.1%), prediabetic status (21.3%), hypertension (17%), and DM (12.1%) were reported among Somali refugees [13]. Diabetes, obesity, and dyslipidemia rates were found to be significantly higher in Somalis than in other ethnic groups in another study comparing Somali refugees to Middle Eastern and Russian refugees [10]. Our results have suggested that adult Somali inhabitants with T2DM have poor glycemic control and a higher incidence of dyslipidemia subgroups. Due to the country’s protracted civil war, the inability to provide appropriate treatment and follow-up for chronic diseases has resulted in a disregard for the importance of education, social awareness, and lifestyle changes. It should also be acknowledged that poverty and war are serious public health issues that have a negative impact on treatment and survival outcomes [18–20].

We have found the mean HbA1c value of 9.7 ± 2.2, which is far from the current recommended target levels for diabetes regulation [21]. Moreover, females had nearly twice the rate of T2DM as males (66.5% vs. 33.5%). This finding is consistent with previous research revealing that women have higher prevalence rate of diabetes, particularly in third-world countries [22, 23]. This gender disparity against women could be attributed to the following reasons; (*a*) receiving less education due to religious and socio-cultural differences, (*b*) taking less advantage of healthcare opportunities, (*c*) Living apart from social life, not being conscious of health, self-care, follow-up, and treatment options, and thereby being more vulnerable to environmental factors.

In our investigation, the rate of total dyslipidemia among T2DM patients was found to be enormously high (92.8%). This was, however, close to the proportions of nations with similar degrees of development in the available literature. The rate, for example, was reported as 95% in Tanzanian study [24] and 89% from South Africa [25]. Another study from Nigeria found the rate to be 69.3% [26]. On the contrary, the frequency of dyslipidemia was relatively low in studies from industrialized nations, such as the United States and the United Kingdom [27, 28]. The present study has demonstrated that the frequency of dyslipidemia is impacted by regional socioeconomic conditions, the failure to implement comprehensive health policies, and insufficient follow-up and treatment, as is the case with many chronic illnesses. However, we consider that the poor health implications of many chronic diseases in nations, such as Somalia, which has long had insufficient health infrastructure for several reasons, are avoidable.

Cigarette consumption among Somalis is low, but men consume significantly more than women, which could be attributed to societal norms and religious beliefs [29]. Smoking women, for instance, are stigmatized in Somali society [30]. Furthermore, the current smoking rate among Somali youth in the United States has been reported to be 4.7%, which is similar to our findings [31].

Atherogenic dyslipidemia, which includes hypertriglyceridemia, low HDL-C, and high LDL-C, is linked to the development of microvascular and/or macrovascular disease, as well as poor cardiovascular outcomes [32]. In the multicenter ACCORD trial, the prevalence of atherogenic dyslipidemia was found to be 17%, compared to 24.8% in the present study [33]. Our study’s considerably greater prevalence of atherogenic dyslipidemia may be connected to poor blood glucose control and obesity. These findings add to our understanding of the link between atherogenic dyslipidemia and impaired glucose control in T2DM patients [34].

In the present research, the most often diagnosed subgroups of dyslipidemias were high non-HDL-C (82.8%), followed by high LDL-C (72.6%), high TC (60.2%), and low HDL-C (53.5%), with hypertriglyceridemia being the least common (47.3%). These results are in line with the few reports undertaken in several African nations [23, 35, 36]. Other studies from the region [36, 37] have found LDL-C dominance in combined dyslipidemias. However, the distributions of dyslipidemia patterns in diabetic individuals have varied between publications. These differences may be related to the research populations' geographical ethnic/genetic variety, dyslipidemia definitions, study methodology, and degree of glycemic control. LDL-C has so distinguished itself as a more atherogenic and powerful cardiovascular risk factor than other dyslipidemia components. As a result, LDL-C is still the primary target of lipid-lowering medication [38]. In our study, such a high prevalence of dyslipidemia in patients with T2DM is not coincidental; it is referred to as diabetic dyslipidemia. Although the exact mechanism of dyslipidemia in T2DM patients is uncertain, insulin resistance may impair lipoprotein lipase activity, which is thought to be crucial in the onset of diabetic dyslipidemia. It may also cause a delay in the clearance of triglyceride-rich lipoproteins, thereby resulting in higher levels of LDL-C, TG, and lower levels of HDL-C [39]. Because of the high LDL-C rates in both isolated and combined dyslipidemia patterns, we should underline the importance of prescribing statins as the primary treatment choice in our study.

The present study contains several drawbacks. First off, although being conducted in the best-equipped and largest institution in Somalia, the research does not have a design that is representative of the entire nation. Second, since the study was cross-sectional, a causal connection could not be proven. Third, there is a lack of knowledge on factors including the ratio of rural to urban areas, educational attainment, physical activity, length of DM, and alcohol use that may be linked to diabetic dyslipidemia. Another significant shortcoming is the country’s present economic position, which makes it difficult to study additional in-depth laboratory tests, such as apoproteins, lipoprotein(a), and lipoprotein-associated phospholipase A2, which may be connected to dyslipidemia.

## Conclusions

Our study is the first comprehensive report describing the pattern and distribution of dyslipidemia among T2DM patients in Somalia. We have also identified much lower rates of blood glucose control than indicated goal values, as well as high prevalence of dyslipidemia, particularly atherogenic dyslipidemia. The epidemiological consequences of diabetic dyslipidemia in Somalia point to the need for health authorities to develop and put into action new policies that could increase public awareness by altering lifestyle choices and providing patients with T2DM with the best management and care possible.

## Supplementary Information

**Additional file 1: Table S1.** Multivariable logistic regression analyses of the male population for predictors of the different types of dyslipidemia. **Table S2**. Multivariable logistic regression analyses of the female population for predictors of the different types of dyslipidemia.

## Data Availability

On reasonable request, the corresponding author will provide the data supporting the study’s conclusions.

## Abbreviations

DM: Diabetes mellitus
HDL-C: High-density lipoprotein cholesterol
LDL-C: Low-density lipoprotein cholesterol
TC: Total cholesterol
TG: Triglycerides
T2DM: Type 2 diabetes mellitus
NCEP–ATP: National Cholesterol Education Program–Adult Treatment Panel
