# Supplementary material for: The pattern of dyslipidemia among Somali type 2 diabetic patients: a cross-sectional study
Source: Eur J Med Res. 2022 Nov 20;27:253. doi: 10.1186/s40001-022-00882-x (PMC9677666; doi:10.1186/s40001-022-00882-x)
Supplement: Supplementary file 1 — Additional file 1: Table S1. Multivariable logistic regression analyses of the male population for predictors of the different types of dyslipidemia. Table S2. Multivariable logistic regression analyses of the female population for predictors of the different types of dyslipidemia. [file 40001_2022_882_MOESM1_ESM.docx]

| **Supplementary Table S1. Multivariable logistic regression analyses of the male population for predictors of the different types of dyslipidemia**   \| **Variables** \| **High-TG**  **OR (95% CI)** \| ***p*-value** \| **High-TC**  **OR (95% CI)** \| ***p*-value** \| **Low-HDL-C**  **(OR) (95% CI)** \| ***p*-value** \| **High-LDL-C** \| **High-Non-HDL-C** \| \| \|  \|  \|  \|  \| \| --- \| --- \| --- \| --- \| --- \| --- \| --- \| --- \| --- \| --- \| --- \| --- \| --- \| --- \| --- \| \| **OR (95% CI)** \| ***p*-value** \|  \| **OR (95% CI)  *p*-value** \| \| \|  \|  \| \| **Age** \| 1.02 (0.98–1.06) \| 0.343 \| 1.01 (0.97–1.06) \| 0.572 \| 1.02 (0.98–1.07) \| 0.286 \| 0.99 (0.93–1.04) \| 0.639 1.05 (0.99–1.13) 0.116 \| \| \|  \|  \|  \|  \| \| **BMI** \| 1.01 (0.86–1.18) \| 0.945 \| 1.01 (0.85–1.21) \| 0.881 \| 1.02 (0.86–1.20) \| 0.845 \| 0.92 (0.73–1.16) \| 0.489 0.98 (0.77–1.25) 0.885 \| \| \|  \|  \|  \|  \| \| **Central obesity** \| 0.45 (0.11–1.85) \| 0.270 \| 1.36 (0.30–6.13) \| 0.685 \| 0.47 (0.11–2.01) \| 0.309 \| 4.14 (0.60–28.48) \| 0.149 1.15 (0.13–10.12) 0.900 \| \| \|  \|  \|  \|  \| \| **Proteinuria** \| 1.91 (0.62-5.87) \| 0.261 \| 1.59 (0.46–5.50) \| 0.465 \| 2.61 (0.83–8.28) \| 0.103 \| 2.04 (0.35–12.09) \| 0.432 6.60 (0.52–83.34) 0.144 \| \| \|  \|  \|  \|  \| \| **Fasting plasma glucose** \| 1.00 (0.99–1.00) \| 0.190 \| 1.00 (1.00–1.01) \| **0.032** \| 1.00 (0.99–1.00) \| 0.571 \| 1.00 (1.00–1.01) \| 0.219 1.01 (0.99–1.01) 0.104 \| \| \|  \|  \|  \|  \| \| **Poor glycemic control** \| 1.70 (0.32–9.07) \| 0.535 \| 0.65 (0.11–3.82) \| 0.636 \| 2.27(0.38-13.64) \| 0.371 \| 0.85 (0.10–7.56) \| 0.884 0.89 (0.07-12.11) 0.929 \| \| \|  \|  \|  \|  \| \| **Creatinine** \| 0.91 (0.57–1.44) \| 0.682 \| 0.44 (0.15–1.28) \| 0.133 \| 1.36 (0.65-2.85) \| 0.410 \| 0.74 (0.41-1.33) \| 0.319 0.86 (0.46–1.59) 0.622 \| \| \|  \|  \|  \|  \| \| **Hypertension** \| 1.33 (0.30–6.02) \| 0.709 \| 0.37 (0.08-1.75) \| 0.210 \| 0.98 (0.21–4.56) \| 0.984 \| 0.60 (0.05–7.00) \| 0.686 0.37 (0.07–1.88) 0.228 \| \| \|  \|  \|  \|  \| \| **Hs-CRP** \| 1.00 (0.97–1.03) \| 0.878 \| 0.97 (0.94–1.00) \| 0.068 \| 1.02 (0.98–1.05) \| 0.298 \| 0.97 (0.93–1.02) \| 0.199 0.95 (0.91–1.00) 0.070 \| \| \|  \|  \|  \|  \|     Values are presented as OR (95% CI). OR = Odds ratio, CI = Confidence interval.  Coding of categorical variables-Gender: female = 0, male = 1; Smoking: non-smoker = 0, current smoker = 1; Proteinuria: absent = 0, present = 1; Glycemic control: good (HbA1c% ≤7) =0, poor (HbA1c% >7) =1; Hypertension: normotensive = 0, hypertensive = 1; Fasting plasma glucose, Age, BMI, Hs-CRP, Creatinine, waist circumference were continuous variables. |
| --- | --- | --- | --- | --- | --- | --- | --- | --- | --- | --- | --- | --- | --- | --- | --- | --- | --- | --- | --- | --- | --- | --- | --- | --- | --- | --- | --- | --- | --- | --- | --- | --- | --- | --- | --- | --- | --- | --- | --- | --- | --- | --- | --- | --- | --- | --- | --- | --- | --- | --- | --- | --- | --- | --- | --- | --- | --- | --- | --- | --- | --- | --- | --- | --- | --- | --- | --- | --- | --- | --- | --- | --- | --- | --- | --- | --- | --- | --- | --- | --- | --- | --- | --- | --- | --- | --- | --- | --- | --- | --- | --- | --- | --- | --- | --- | --- | --- | --- | --- | --- | --- | --- | --- | --- | --- | --- | --- | --- | --- | --- | --- | --- | --- | --- | --- | --- | --- | --- | --- | --- | --- | --- | --- | --- | --- | --- | --- | --- | --- | --- | --- | --- | --- | --- | --- | --- | --- | --- | --- | --- | --- | --- | --- | --- | --- | --- | --- | --- | --- | --- | --- | --- | --- | --- | --- | --- | --- | --- |

| **Supplementary Table S2. Multivariable logistic regression analyses of the female population for predictors of the different types of dyslipidemia**   \| **Variables** \| **High-TG**  **OR (95% CI)** \| ***p*-value** \| **High-TC**  **OR (95% CI)** \| ***p*-value** \| **Low-HDL-C**  **(OR) (95% CI)** \| ***p*-value** \| **High-LDL-C** \| **High-Non-HDL-C** \| \| \|  \|  \|  \|  \| \| --- \| --- \| --- \| --- \| --- \| --- \| --- \| --- \| --- \| --- \| --- \| --- \| --- \| --- \| --- \| \| **OR (95% CI)** \| ***p*-value** \|  \| **OR (95% CI)  *p*-value** \| \| \|  \|  \| \| **Age** \| 1.03 (1.00–1.06) \| **0.046** \| 0.99 (0.97–1.02) \| 0.497 \| 1.01 (0.99–1.04) \| 0.335 \| 0.99 (0.96–1.03) \| 0.591 0.97 (0.94–1.00) 0.077 \| \| \|  \|  \|  \|  \| \| **BMI** \| 1.28 (1.11–1.47) \| **0.001** \| 1.13 (0.99–1.28) \| 0.068 \| 0.92 (0.82–1.04) \| 0.182 \| 1.14 (0.97–1.34) \| 0.106 1.17 (0.98–1.39) 0.080 \| \| \|  \|  \|  \|  \| \| **Proteinuria** \| 1.90 (0.85-4.22) \| 0.116 \| 0.93 (0.41–2.08) \| 0.857 \| 0.23 (0.10–0.51) \| **<0.001** \| 1.35 (0.49–3.70) \| 0.566 1.06 (0.38–2.99) 0.912 \| \| \|  \|  \|  \|  \| \| **Fasting plasma glucose** \| 1.01 (1.00–1.01) \| **0.002** \| 1.00 (0.99–1.00) \| 0.198 \| 1.00 (0.99–1.00) \| 0.916 \| 1.00 (1.00–1.01) \| 0.158 1.00 (1.00–1.01) 0.119 \| \| \|  \|  \|  \|  \| \| **Poor glycemic control** \| 0.87 (0.34-2.25) \| 0.776 \| 0.26 (0.08–0.86) \| **0.027** \| 0.92 (0.36-2.33) \| 0.862 \| 0.53 (0.14–1.98) \| 0.345 0.64 (0.18-2.31) 0.495 \| \| \|  \|  \|  \|  \| \| **Creatinine** \| 0.99 (0.72–1.35) \| 0.928 \| 0.45 (0.22–0.94) \| **0.033** \| 1.36 (0.93-1.99) \| 0.110 \| 0.84 (0.60–1.17) \| 0.295 0.61 (0.38–0.96) **0.032** \| \| \|  \|  \|  \|  \| \| **Hypertension** \| 1.18 (0.42–3.29) \| 0.756 \| 1.28 (0.49-3.35) \| 0.617 \| 0.64 (0.24–1.73) \| 0.384 \| 2.47 (0.83–7.37) \| 0.106 3.60 (1.13–11.43) **0.030** \| \| \|  \|  \|  \|  \| \| **Hs-CRP** \| 1.00 (0.98–1.03) \| 0.706 \| 0.98 (0.96–1.00) \| 0.052 \| 1.01 (0.99–1.03) \| 0.530 \| 0.97 (0.95–1.00) \| **0.023** 0.99 (0.97–1.02) 0.488 \| \| \|  \|  \|  \|  \|     Values are presented as OR (95% CI). OR = Odds ratio, CI = Confidence interval.  Coding of categorical variables-Gender: female = 0, male = 1; Smoking: non-smoker = 0, current smoker = 1; Proteinuria: absent = 0, present = 1; Glycemic control: good (HbA1c% ≤7) =0, poor (HbA1c% >7) =1; Hypertension: normotensive = 0, hypertensive = 1; Fasting plasma glucose, Age, BMI, Hs-CRP, Creatinine, waist circumference were continuous variables. |
| --- | --- | --- | --- | --- | --- | --- | --- | --- | --- | --- | --- | --- | --- | --- | --- | --- | --- | --- | --- | --- | --- | --- | --- | --- | --- | --- | --- | --- | --- | --- | --- | --- | --- | --- | --- | --- | --- | --- | --- | --- | --- | --- | --- | --- | --- | --- | --- | --- | --- | --- | --- | --- | --- | --- | --- | --- | --- | --- | --- | --- | --- | --- | --- | --- | --- | --- | --- | --- | --- | --- | --- | --- | --- | --- | --- | --- | --- | --- | --- | --- | --- | --- | --- | --- | --- | --- | --- | --- | --- | --- | --- | --- | --- | --- | --- | --- | --- | --- | --- | --- | --- | --- | --- | --- | --- | --- | --- | --- | --- | --- | --- | --- | --- | --- | --- | --- | --- | --- | --- | --- | --- | --- | --- | --- | --- | --- | --- | --- | --- | --- | --- | --- | --- | --- | --- | --- | --- | --- | --- | --- | --- | --- | --- |
